# The occupation space: network structure, centrality and the potential of labor mobility in the French labor market

**DOI:** 10.1007/s41109-022-00453-3

**Published:** 2022-03-14

**Authors:** Charlie Joyez, Catherine Laffineur

**Affiliations:** Université Cote d’Azur, GREDEG, 06560 Valbonne, France

**Keywords:** Network analysis, Occupational mobility, Centrality, C45, E24, J24, R23

## Abstract

This article presents the Occupation Space, a weighted and directed network of occupations built from an extensive database that tracks French workers employment trajectories between 2003 and 2015. In this network, the links between occupations stands for the easiness to switch from one occupation to another that we interpret as being a good proxy for skill proximity between occupations. The article first describes the structural characteristics of the network. We show that some occupations offer workers important redeployment possibilities to other occupations. Then we use information on the centrality of occupations in the network to analyze its correlation with wage premium and unemployment duration. Our results show that the network-based index of centrality is informative of the sources of several labor market outcomes and inequalities.

## Introduction

Workers are at risk to many destabilizing events. Economic downturn, major policy changes, technological breakthrough, increase competition from foreign countries, extreme natural events, and pandemics are all events able to undermine a nation’s performance at any moment in time. When such events arise, workers’ mobility and adaptability is key to survive in a changing environment. However, while several studies examined workers’ mobility across industries,^1^ evidence regarding the redeployment possibilities across occupations is limited, which is surprising considering that occupation mobility is a common event in a worker’s career life,^2^ and considering that the redeployment possibility of workers towards other occupations might have important implications for labor market outcomes.^3^Therefore, we argue that before the causes and effects of worker mobility can be assessed, we need a clear understanding of how occupations are connected to one another and how this connection relates with labor market outcomes.

This assessment is the main contribution of this article. The goal of this research is then to picture occupation mobility into an “Occupation Space” to identify the skill relatedness between occupations. We then estimate the role of workers’ redeployment possibilities on several labor market outcomes including wage premia and unemployment duration.

To be precise, we estimate the proximity between occupations by measuring the likelihood to switch from one occupation to another based on the actual flows between occupations and controlling for a set of characteristics influencing occupation mobility (size, gender, age, and earnings prospects). We interpret large bilateral mobility as the reflect of intense skill-relatedness between occupations. We picture occupation mobility in an Occupation Space, in which nodes represent occupations and the arcs depict the ease of jobs switches between two occupations. We detail the structure of the network and the related communities of jobs.

Then, we estimate whether the position of a worker in this occupation space correlates with several labor market outcomes. We develop a score of outward “centrality” for 269 different occupations. The centrality of an occupation determines its immediate redeployment possibilities towards other occupations in the network. We then estimate the relationship between the workers’ occupation centrality and its hourly wage, unemployment duration and the likelihood to find a job when being unemployed. In line with our theoretical predictions, we find that central occupations allow for higher bargaining power and redeployment possibilities. Specifically, occupation centrality is associated with a significantly higher wage premium, a reduction of unemployment duration and a greater likelihood to find a job when being unemployed.

Our study contributes to several branches of the literature. First, we contribute to the literature seeking the roots of workers mobility (Poletaev and Robinson 2008; Kambourov and Manovskii 2008, 2009; Lalé 2012; Longhi and Brynin 2010). Occupational mobility has been already studied using longitudinal dimension of demographic surveys. Yet existing works mainly focus on identifying the phenomenon and its frequency. Interestingly, evidence show that occupational mobility is weakly correlated to jobs’ (or employers’) switch (Moscarini and Thomson 2007), and that occupational mobility experiences an increasing trend since the 1990s both in the US and in France (Lalé, 2012). This literature examines the heterogeneity of occupational mobility across workers groups of age, gender and education, but does not examine how occupation themselves influence labor mobility through the skill proximity between them. A few studies examined this issue, with notably Gathman and Schönberg (2010) which focuses on the portability of skills from one occupation to another. They show that occupation mobility is influenced by the similarity of task-requirements and conclude that skills are largely transferable across occupations. They show that skill proximity is becoming more important to understand workers flows across occupation. Pohlig (2021) reports that the Great Financial crisis has affected occupational mobility by increasing downward mobility. The current covid19 crisis is therefore an additional motivation to investigate pattern of occupational mobility across workers.

We also contribute to the literature on the sources of labor market inequality, including wage and gender inequality (Manning and Swaffield 2008; Abowd et al. 1999), and offer new perspective on the origins of workers’ bargaining power in the labor market that was mainly associated with education since then (Cahuc et al. 2006). Our result might also be of interest for researchers analyzing the role of occupation space on the transformation of urban areas (Muneepeerakul et al., 2013, Duranton and Puga 2005). Finally, our results might enrich our understanding of the workers at risks of unemployment from automation and globalization (Arntz et al. 2017; Frey and Osborne 2017; Baldwin 2019; Katz and Author 1999). Indeed, a growing body of the literature explains unemployment by a mismatch between unemployed workers and jobs offers skills (Shimer 2007; Şahin et al. 2014). Biased technological progress or trade openness will therefore have varied effect on unemployment risks according to the mobility potential of workers in each occupation. We believe that this research paves the way for future work concerning the role of occupation mobility on the labor market.

The rest of this paper is organized as follows. "The occupation space" section describes the Occupation Space, while "Centrality of occupations" section provides a short description of our measure of occupation centrality. In "The benefits of central occupations" section, we present our main results on how occupation centrality relates with several labor market outcomes. We conclude in the final section and provide ideas for future works.

## The occupation space

### Data and methodology

In order to build the Occupation Space, we use the administrative panel—*Déclaration Annuelles des Données Sociales (DADS Panel)*. The data is built from confidential yearly social-security records, treated and transmitted by the French National Institute for Statistics (INSEE). Administrative records are based on firms’ mandatory report of workers subject to payroll taxes to fiscal authorities. The database covers all firms in the private and public industries. From this administrative record, a panel of individuals born in October is built. Each observation consists of an employer-employee match and reports the sex, age, residence and workplace’s region, yearly real earnings (in 2007 euros) and the number of hours and days worked each year by the individual. Since wages and careers are likely to be affected by personal events such as birth or marriage, we use data enhanced by information from the Permanent Demographic Sample (*échantillon démographique permanent, EDP*). The Permanent Demographic Sample is augmented with variables from the annual census surveys. Currently, more than 1,000,000 individual’s social and professional trajectories are well tracked. This data source gives details on education, marital status, and number of children.

This dataset is very large and sometimes noisy, which is why we apply various cleaning procedures. First, we focus on mainland France and remove overseas territories. Second, as is customary in the literature, we keep workers in private industry besides traineeship and subsidized employment, within working age range (15–65). Third, to focus on meaningful working experiences, we remove observations of occupations held for less than 30 days. Also, workers in the DADS can be identified simultaneously in several positions, we only keep the worker-firm match for which the job spell and salary is the highest, and remove occupations identified as annex ones. Finally, we drop occupations for which less than 10 outward flows were observed, as very small sample are likely to be not representative.

After our cleaning procedure, we end up with a non-balanced-yearly-worker panel that includes 969,348 workers representative of the French workforce employed in 269 occupations with an average of 7.8 years of observations per workers. Job switches are frequent events. Workers hold on average 2.5 different occupations and more than 60% of workers have experienced at least one occupation switch over the period. One reason why we notice some high rates of job switches is because we also capture the ones that occur within the same firms, which represent 53% of occupation mobility. We also notice that the distribution of occupation switch is right skewed, as 50% of the sample only switched once, and over one third of workers (36.24%) remained in the same occupation.

**Fig. 1:**
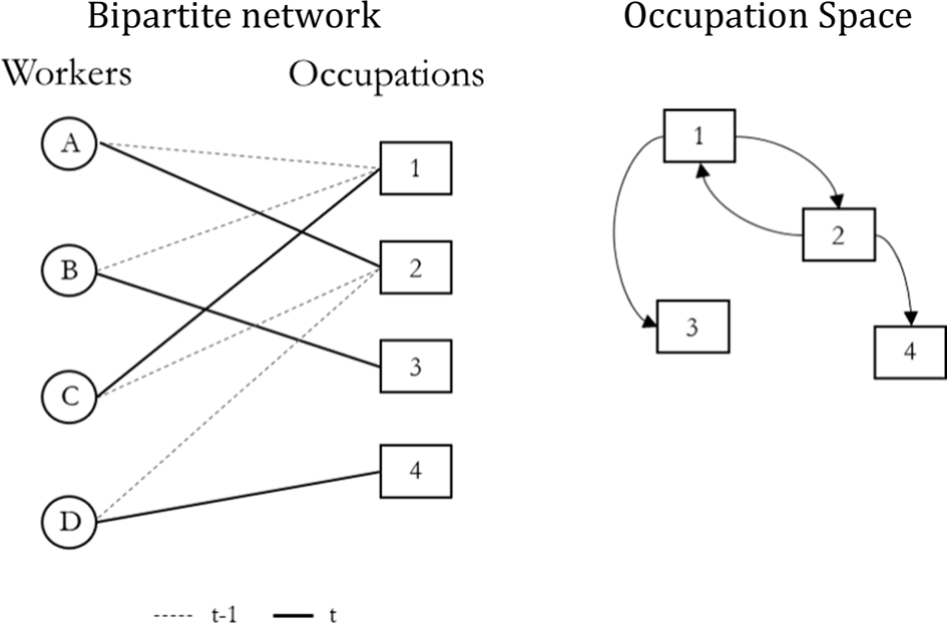
From panel data to network. *Note*: Worker *A* held occupation 1 in *t* − 1 and then occupation 2 at time *t*, generating the directed arrow from node 1 to 2 in the occupation space

We represent the *DADS* panel as an affiliation network between individuals and their occupations (see left panel of Fig. 1). We turn this bipartite network with two types of nodes into a unimodular network through a projection into one of its dimensions only (Zhou et al. 2007).^4^ When projecting our network, we consider the direction of the job switches, and we only link immediately successive occupations.^5^

The resulting network details mobility pattern between occupations, as illustrated in the right panel of Fig. 1.

Clearly, flows between occupations are not sufficient to capture the skill-proximity between occupations because occupation mobility is also determined by workers’ socio-economic characteristics, as it has been largely documented since the 1980s. Namely, educated young men change jobs much more frequently than respectively older workers, women, or non-educated employees (Blumberg 1980; Groes et al. 2014). Occupations switches are also determined (or limited) by local labor demand of firms, with notably a positive influence of the spatial employment density on the probability of job switch (Andersson and Thulin 2013). The realization of job switches opportunities also depends on the current and future earnings (Topel and Ward 1992), such that actual flows give a flawed reflect of actual skill proximity.

Hence, determining whether occupations are related according to the number of flows is insufficient. We need to determine whether the flow is *exceptionally* large compared with a baseline. Therefore, we follow Neffke and Henning (2013) to measure the baseline of occupation mobility. This baseline should reflect our expectations of the size of a labor flow between two occupations based only on some general characteristics of the occupations involved.

More precisely, we define occupation proximity by the following equation:

$${Proximity}_{i,j}=\frac{{F}_{i,j}}{\widehat{{F}_{i,j}}}$$

${F}_{i,j}$ is defined as the number of workers who switch from occupation *i* to occupation j in the same location during the time span^6^ and $\widehat{{F}_{ij}}$ is predicted labor flows, in a “flat world” baseline, i.e. a world where all occupations would be equidistant in skills requirement.

The predicted labor flows $\widehat{{F}_{ij}}$ come from a regression analysis of ${F}_{ij}$ (i.e. total employment from the occupation of origin *i* to the occupation of destination *j*). This regression accounts for the growth of flows during the period of observation, the wage premium of the switch, the average age in the occupation of destination and origin and the share of male workers in the occupation of origin and destination. We also included two variables controlling whether both occupations are mainly in rural or urban commuting zones, to account for a geographic restriction of occupational mobility. An occupation is said to be urban if its share in the 10 biggest French metropolitan areas is higher than in overall France, and rural otherwise. Because raw labor flows are non-negative by nature but characterized by a large number of zeros (unobserved trajectories), we follow Neffke and Henning (2013) and opt for a zero-inflated negative binomial (ZINB) regression model. The resulting estimated coefficients are used to construct the predicted values $\widehat{{F}_{ij}}$ (see Appendix 1 for further details on the ZINB model).

By dividing real flows by expected flows according to size, earning opportunities or socio-composition of occupations, and their geographic distribution, we measure the proximity of occupations that is independent from these observable characteristics. Among unobserved characteristics that determine workers’ flows, we believe that a large share depends on the proximity of skills requirement, as shows the literature on skills matching and jobs search mentioned in introduction. We follow Neffke and Henning (2013), Neffke et al. (2017) and assume our ${Proximity}_{i,j}$ index to be a proxy of skill-relatedness across occupation. We then only keep edges with a value of ${Proximity}_{i,j}>1$, for all edges’ weight to be interpreted similarly as reflecting occupation relatedness.^7^

**Fig. 2:**
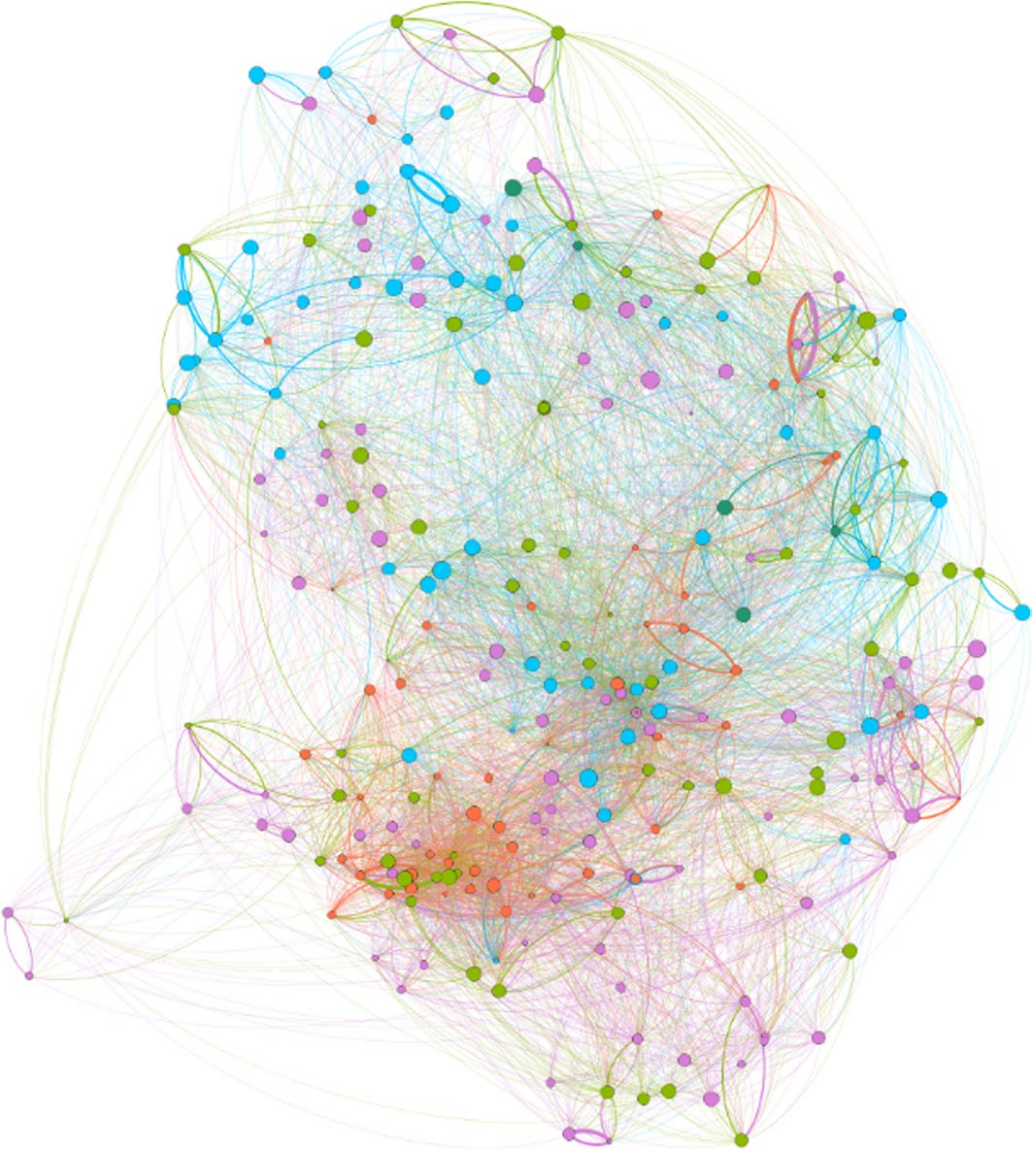
The French Occupation Space. This graph has been realized with *Gephi* software using OpenOrd algorithm (Martin et al. 2011). The nodes’ sizes are proportional to their outward centrality, and their color correspond to PCS-1digit communities classes. Edges’ sizes are proportional to their weights. Only top 50% of existing edges were represented

We measure ${Proximity}_{i,j}$ indices for each of the 269 × 268 = 72,092 combinations of 269 occupations at the 4-digit level of the French PCS-ESE occupation classification. Using *Proximity*_*ij*_ scores as edges’ weights, we built our final network depicted in Fig. 2 and labeled it the “Occupation Space” referring to the “product space” of Hidalgo et al. (2007).^8^

### Network characteristics of the occupation space

The Occupation Space is a weighted and directed network, made of 269 nodes, with a density of 0.175. This result shows that approximately one sixth of potential job switches are more common than the ones predicted by measures from socio-economic composition of occupations. The arcs’ weights correspond to the excess ratio of flows and reflect the easiness to switch from one occupation to another. Network-wide indexes detail the overall connectivity structure of the Occupation Space, informing us on the average relatedness of occupations, and the distribution of skill-proximity across them. First, it is worth noting that the Occupation Space is only made of one largest component including all nodes without isolated sub-networks. In order to facilitate the interpretation of the Occupation Space’s characteristics, we compare it to a null model, made of 1000 draws of directed Erdos–Renyi networks of the same size, density, and total weights, but where all linkages have the same probability to exist. Significant deviations from the null models are therefore perceived as singular characteristics of our skill-proximity network. Table 1 compares the main network-wide indexes from the null model (displaying its 95% confidence interval) and from the Occupation Space.

**Table 1:** Comparison of network structure: random versus occupation space

Index	95% CI null model	Occupation space
Diameter	[3;3]	3
average path	[1.825;1.825]	1.98
clustering coefficient	[0.1801; 0.1830]	0.176
Disparity	[0.0291; 0.0298]	0.031
Degree centralization	[0.0528; 0.0865]	0.188
Strength centralization (weighted)	[0*.*0028; 0.0045]	0.0132
Reciprocity	[0.1110;0.1229]	0.820

The Occupation Space displays some interesting features about the mobility potential on the labor market. First, the potential of mobility across occupations is larger than commonly thought. The average outward degree is 46.8, indicating that each occupation offers on average a large range of “close” redeployment opportunities, and only 10% of occupations gives a preferential access to less than 24 others. Of course, all neighbors are not equally close, but the average *disparity* of edges’ weight across outward ties is higher than in a null model, reflecting a relative balanced distribution of outward mobility potential across “close” occupations.^9^ Barthélemy et al. (2005) suggest using reverse value of the disparity index to reflect the number of dominant neighbors. When following their suggestion, we find on average 33 substantial redeployment possibilities from each occupation.

The second information is more intuitive and shows that the mobility between occupations tends to reciprocate, with a reciprocity above 7 times higher than the random counterfactual. But potential job mobility is not transitive, as indicated by the lower clustering coefficient than the one displayed in random networks (either weighted or not). This can be explained by different subsets of skills in common between two neighboring occupations, preventing them to be direct neighbors themselves.

Third, the occupation space is not exactly structured as a small-world network (Watts and Strogatz 1998), as it has a lower clustering and similar average path than the one displayed in the null networks. Contrariwise, centralization is similar than the one in a small-world network. Our network has a Freeman (1978) centralization index higher than in the one measured from the random networks (from both weighted and unweighted dimensions). This centrality reflects an easier access to a wider range of occupations. Therefore, redeployment possibilities are unevenly distributed across occupations.^10^ Workers employed in more central occupations have more redeployment possibilities towards other occupations. We further investigate this dimension in the next section.

## Centrality of occupations

### Measuring centrality

**Fig. 3:**
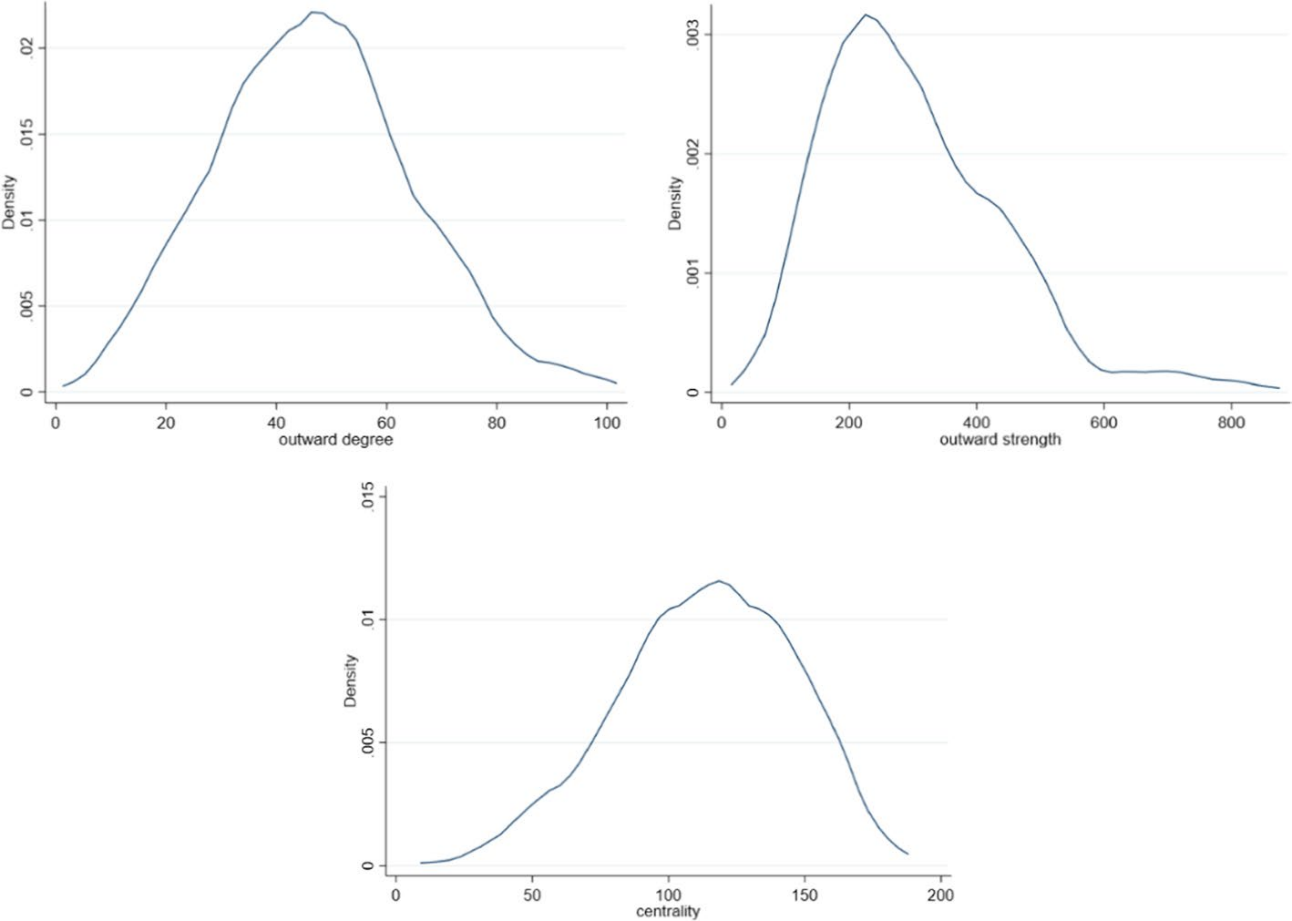
Distribution of nodes centrality indexes. *Note*: The Figure displays the distribution of nodes (occupations) according to several outcomes: outward degree (upper left), outward strength (upper right) and centrality (lower panel)

Centrality is a common notion in network analysis. It refers to the relative importance of each node in the network. Various indexes can be used to capture centrality. In the occupation space, we refer to centrality as the relative redeployment possibilities provided by each occupation. We will therefore focus on outward linkages only to assess the centrality of one node. In addition, we aim at capturing *immediate* redeployment possibilities, as they are the ones that should matter the most for labor market outcomes. We therefore consider first-order connectivity to compute centrality and not higher-order ones, such as closeness or betweenness, that respectively capture average distance and position on shortest path between nodes. Outward degree—the number of outgoing neighboring nodes—is an obvious measure of direct connectivity, yet it does not capture the weighted dimension of the network. To capture this additional dimension, we follow by Opsahl et al. (2010), and define centrality as follows^11^:

1 $$Ci = Ki^{{0.{5}}} * Si^{{0.{5}}}$$

where *k*_*i*_ is the outward *degree* of occupation *i* (i.e., the number of occupations that one occupation gives easier access to) and *s*_*i*_ is the outward *strength* of the occupation (i.e., the sum of its outward edges’ weights). Figure 3 shows the distribution of outward degree, strength, and centrality as defined above. The centrality index and outward degree is somehow normally distributed, but it is still more concentrated than the ones from random networks such as the Freeman network.

### Central occupations

Table 2 reports the top five or most central occupations and the five least central or peripheral ones. Industrial supervisors and engineers are the occupations offering the largest set of outgoing redeployment. It is worth noting that the top 5 central occupations include two “intermediate professions” as classified by French statistical institute (PCS 4 at the 1 digit level), two upper intellectual ones (PCS 3), and one specialized worker (PCS 6). These nodes are connected to industrial occupations, but also to executive or supervision occupations outside manufacturing industries. Regarding the least central occupations, the “employees” category (PCS 5) is over-represented with three out of five occupations. They correspond to low qualified occupations (hairdressers), or to other very specialized occupations that require very with specific skills (airlines stewards, taxi drivers, childcare). Consequently, these occupations offer fewer redeployment possibilities.^12^

**Table 2:** Most and least central occupations

Centrality rank	Occupation code	Occupation	Out degree	Out strength	Centrality
1	483a	Supervisors in mechanical engineering, metalworking	46	694.2619	178.7066
2	387e	Engineers and managers of maintenance, upkeep and new works	69	454.6431	177.1168
3	386b	Engineers and study executives, research and development of energy distribution, water	60	521.8799	176.9542
4	628d	Skilled setters of manufacturing equipment (excluding metalworking and mechanical)	36	835.0168	173.3799
5	484b	Supervisors in manufacturing: metallurgy, heavy materials and other processing industries	50	595.401	172.54
…					
265	562b	Salaried hairdressers	26	70.49376	42.81165
266	642a	taxi drivers	9	183.2547	40.61147
267	431f	Nurses in general care, salaried	14	106.7873	38.66552
268	526c	Childcare auxiliaries	12	96.81557	34.085
269	546d	Stewards	6	55.04745	18.17374

## The benefits of central occupations.

In this section, we analyze the correlates between occupation centrality in the occupation network and two labor market outcomes: wage premium and unemployment duration. Although this section is descriptive in nature and does not aim to assess causal relationships, to our knowledge, such an undertaking, to describe the role of occupation centrality on labor market outcomes, has not been previously done in the literature. Because systematic evidence on the role of occupation centrality is scarce, our view is that even this simple descriptive exercise will contribute to the literature on labor market inequality.

### Data

We run our analysis using a representative sample of French employed and unemployed workers from the French Labor Force Survey (LFS) over the period 2003–2012. The LFS is a continuous survey providing quarterly data. Participation is compulsory and it covers private households in mainland France. All individuals in the household older than 15 are surveyed. Topics covered by the LFS concern employment, unemployment, underemployment, hours of work, wages, duration of employment and unemployment (length of service), discouraged workers, industry, occupation, status in employment, education/qualification, and other jobs. The French LFS provides the occupation for each employed individual among a list of 350 possible occupations according to the French PCS-ESE classification. We merged this LFS with the centrality data computed on the 269 occupations detailed above and drop occupations previously discarded. The final labor force survey considered is made of 1,097,319 observations of 259,631 distinct French workers.

### Workers’ characteristic and centrality of occupations.

**Fig. 4:**
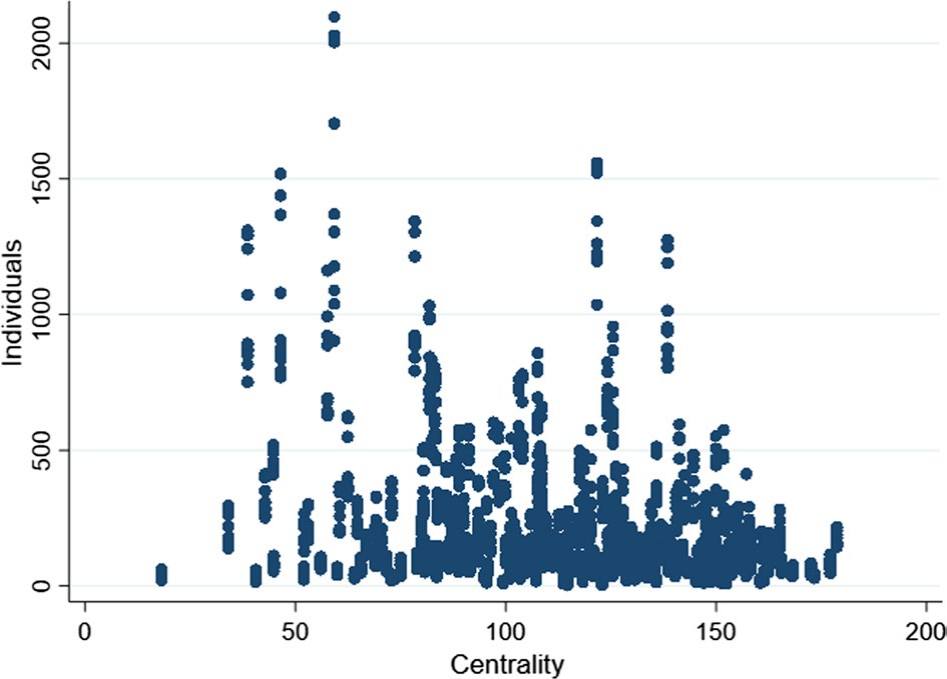
Centrality and number of workers by occupations. *Note*: Each dot corresponds to a particular occupation. For each occupation we report its density (number of workers) and centrality in the network

The potential of job mobility is unevenly distributed on the labor market. Occupations that are the least central, i.e. with fewer redeployment possibilities also employ a large portion of the workforce (Fig. 4).

Next, we report the average centrality of occupations according to several socio-demographic characteristics Table 3 shows that higher educated workers are employed in more central occupations, suggesting that higher education allows to develop several skills that can be transferable to many occupations. We also find that men are employed in substantially more central occupation than women on average, while young workers are employed in less central occupations. In the next section we add these characteristics as control variables.

**Table 3:** Average centrality according to socio-demographic characteristics

	Average centrality (in log)
Overall sample	105.5
*Diploma*	
Master/Ph.D.	123.9*
Some college	108.6*
Upper general high-school	105.9
Upper technical high-school	102.2*
Lower high-school	100.8*
No diploma	98.5*
*Gender*	
Women	114.9*
Men	94.2*
*Age*	
< 30	102.6*
30–39	107.6
40–49	106.4
50–60	105.1

The asterisk indicate the significance of differences in mean compared to overall average

### Methodology

Our empirical strategy has two main goals. First, we aim to establish whether French workers in central occupations outperform their counterparts in less connected occupations in terms of hourly wages (Eqs. 2 and 3 below). Second, we investigate the relationship between the centrality of an occupation and the worker’s unemployment duration (Eqs. 4 and 5). We hypothesize that central occupations that offer workers’ larger redeployment possibilities should provide workers a greater bargaining power and a lower risk of long-time unemployment.

We start by estimating the usual wage premia with a Mincer equation. We regress wage according to the individual’s position in the Occupation Space. Specifically, we run a linear regression (OLS) on the pooled cross section sample, following econometric model:

2 $${Wage}_{i}={\beta }_{1}{Centrality}_{i}+{{\beta }_{2}X}_{i}+{{\beta }_{3}\delta }_{t}+ {\varepsilon }_{i}$$

where our dependent variable ${Wage}_{i}$ characterizes the log of worker *i*’s hourly net wage including bonuses and advances. ${Centrality}_{i}$ refers to the centrality of worker *i's* occupation, as detailed in Eq. (1) and is measured in log. ${X}_{i}$ represents a set of control variables that accounts for gender (which is a dummy that takes the value of one if the worker is a women), for diploma (we define 6 categories of education: (i) higher degree, master, bachelor or PhD, (ii) some college, up to 2 years, (iii) upper high-school general diploma, (iv) upper high-school technical diploma, (v) lower high-school diploma, (vi) no diploma),^13^ age, and seniority within employing firm. We add yearly fixed effects (${\delta }_{t}$) to capture for time specific shocks on wages (e.g. recession in 2009). Finally, we exclude seasonal workers and apprentices, to exclude from our sample workers that are by nature more mobile than other workers for other reasons than the centrality of their occupation.^14^

In a robustness specification, we make use of the pseudo-panel nature of our data to run specification (2) with a fixed-effect model on panel data.^15^ We run the following specification:

3 $${Wage}_{it}={\beta }_{1}{Centrality}_{it}+{{\beta }_{2}X}_{i}+{{\beta }_{3}\delta }_{i}+{{\beta }_{4}\delta }_{t}+ {\varepsilon }_{it}$$

where ${\delta }_{i}$ represent a set of individual fixed effects. This results in dropping the gender variable in ${X}_{i}$, because it is invariant over time, and therefore collinear with our fixed effects. Yet Other control variables of ${X}_{i}$ such as education, age and seniority are still included.

In a second step, we estimate the relationship between the centrality of the last occupation held and unemployment duration for unemployed people in our sample. We proceed as for Eqs. (2) and (3) by first reporting estimation using OLS on cross-section data (Eq. 4) and then by reporting the results of a specification with fixed effect model on panel-data (Eq. 5).

4 $${Unemployment duration}_{i}={\beta }_{1}{Centrality}_{i}+{\beta }_{2}{X}_{i}+{{\beta }_{3}\delta }_{t}+ {\varepsilon }_{i}$$

5 $${Unemployment duration}_{it}={\beta }_{1}{Centrality}_{it}+{\beta }_{2}{X}_{it}+{{\beta }_{3}\delta }_{i}+{{\beta }_{4}\delta }_{t}+ {\varepsilon }_{it}$$

where unemployment duration is the time spent in unemployment in months. The control variables in specification (4) and (5) are similar to the ones described in specification (2) and (3) respectively, except that we remove seniority as it is irrelevant for unemployed.

### Results

Table 4 reports the results of specifications (2) and (3). Our results show that the diversity of potential opportunities (measured with outward degree) and the proximity between occupations (measured with outward strength) matter in determining the wage premium. This result confirms the necessity to use a centrality approach combining these two dimensions. When looking at the results from centrality (columns (3) and (4)), our results confirm our hypothesis that central occupation offers a wage premium. Precisely, when controlling for education and socio-demographic characteristics, a 1% increase in the centrality of an occupation increases hourly wage by 0.20%. This positive relationship is probably the consequence of a higher bargaining power from workers with large redeployment possibilities.

**Table 4:** Correlation between occupation centrality and wage premia

	Dependent variable: Hourly Wage			
	OLS	OLS	OLS	OLS-FE
	(1)	(2)	(3)	(4)
Outward degree	0.045	–	–	–
	(19.72)***	–	–	–
Outward strength	–	0.229	–	–
	–	(96.11)***	–	–
Centrality	–	–	0.203	0.064
	–	–	(70.28)***	(3.83)***
	–	–		
Age	0.006	0.006	0.006	0.028
	(52.83)***	(52.05)***	(52.12)***	(23.63)***
Diploma: (ref: higher diploma)				
Some college	− 0.230	− 0.201	− 0.202	− 0.111
	(59.13)***	(52.44)***	(52.08)***	(3.34)***
Upper general high-school	− 0.464	− 0.410	− 0.432	− 0.181
	(124.81)***	(110.51)***	(116.08)***	(4.92)***
Upper technical high-school	− 0.610	− 0.532	− 0.564	− 0.352
	(177.24)***	(152.60)***	(161.93)***	(8.37)***
Lower high-school	− 0.634	− 0.558	− 0.590	− 0.645
	(137.10)***	(120.78)***	(127.42)***	(13.77)***
No diploma	− 0.828	− 0.735	− 0.778	− 0.525
	(223.43)***	(194.55)***	(207.30)***	(11.16)***
Female (ref: male)	− 0.339	− 0.284	− 0.298	
	(163.70)***	(136.35)***	(140.65)***	
Seniority	0.001	0.001	0.001	0.000
	(111.18)***	(99.86)***	(108.26)***	(1.07)
constant	7.244	6.118	6.433	6.172
	(664.51)***	(414.95)***	(421.94)***	(32.83)***
Year fixed effects	Yes	Yes	Yes	Yes
*R*^2^	0.30	0.32	0.31	0.01
*N*	277,479	277,479	277,479	277,479

This table shows estimate from OLS and FE-OLS regressions of Eq. (2) and (3). The outcome variable is hourly wage. Column (1) shows estimates with outward degree. Column (2) reports estimates with outward strength. Column (3) shows estimates with Opsahl et al. (2010) Centrality index. Column (4) adds individual fixed effects to the estimation reported in column (3). Standard erors are clustered at the workers level

*p < 0.1; **p < 0.05; ***p < 0.01

Our results further show in Table 5 that the centrality of an occupation is negatively correlated with unemployment duration. A small increase in centrality of an occupation reduces unemployment spell by 13%. The result hold when controlling for individual fixed effects, with a smaller effect on hourly wage and on the unemployment length. Precisely, when controlling for workers fixed effects, we show that a 1% increase in centrality raises hourly wage by 0.06% and reduces unemployment spell by 26%. Overall, our results confirms that our index of centrality in the occupation space is a good proxy for workers’ bargaining power and outside options, which can be beneficial for them by increasing net wage and reducing unemployment duration.

**Table 5:** Correlation between last occupation centrality and unemployment duration

	Dependent variable: unemployment length			
	OLS	OLS	OLS	OLS-FE
	(1)	(2)	(3)	(4)
Outward degree	− 0.021	–	–	–
	(1.29)	–	–	–
Outward strength	–	− 0.159	–	–
	–	(9.16)***	–	–
Centrality	–	–	− 0.137	− 0.264
	–	–	(6.46)***	(1.89)*
Age	0.008	0.008	0.008	0.785
	(14.27)***	(15.00)***	(14.51)***	(48.13)***
Diploma: (ref: higher diploma)				
Some college	− 0.058	− 0.069	− 0.064	− 0.100
	(2.02)**	(2.42)**	(2.25)**	(0.24)
Upper general high-school	0.007	− 0.022	− 0.008	− 0.567
	(0.28)	(0.88)	(0.33)	(1.11)
Upper technical high-school	0.059	0.020	0.037	− 0.515
	(2.57)**	(0.86)	(1.57)	(0.87)
Lower high-school	0.116	0.078	0.097	− 0.476
	(4.14)***	(2.76)***	(3.44)***	(0.77)
No diploma	0.194	0.147	0.168	− 0.576
	(8.39)***	(6.23)***	(7.16)***	(0.94)
Female (ref: male)	0.011	− 0.011	− 0.004	
	(0.88)	(0.88)	(0.36)	
Seniority	0.001	0.001	0.001	0.000
	(111.18)***	(99.86)***	(108.26)***	(1.07)
constant	1.069	1.861	1.643	− 24.584
	(14.00)***	(17.92)***	(15.00)***	(24.93)***
Year fixed effects	Yes	Yes	Yes	Yes
*R*^2^	0.02	0.03	0.03	0.20
*N*	25,502	25,502	25,502	25,502

This table shows estimate from OLS and FE-OLS regressions of Eqs. (2) and (3). The outcome variable is unemployment duration. Column (1) shows estimates with outward degree. Column (2) reports estimates with outward strength. Column (3) shows estimates with Opsahl et al. (2010) Centrality index. Column (4) adds individual fixed effects to the estimation reported in column (3). Standard erors are clustered at the workers level

*p < 0.1; **p < 0.05; ***p < 0.01

## Conclusion

This article builds an Occupation Space, represented by a network of occupations that captures the unobservable skill-proximity between 269 different occupations stemming from French labor market data. The Occupation Space maps the bilateral likelihood of jobs switches based on a weighted measure of occupation mobility. The resulting network displays some interesting feature about the actual potential mobility of workers across occupations. The uneven distribution of connectivity in variety and depth of linkages allows some central nodes to offer larger redeployment possibilities to other occupations, which should have some consequences on labor market outcomes. We test this assumption by analyzing the consequences on wages and unemployment length. Our results show that occupation centrality correlates with workers’ wage premium and fosters the return to employment by reducing unemployment duration. These findings suggest that the centrality of an occupation in the Occupation Space is a good proxy for workers’ bargaining power, leading to a wage premium and lower unemployment duration compared with workers in less central occupations. These findings could help identifying the occupations and activities where continuous education and skill training should be prioritized, to reduce the vulnerability of workers, and to foster their redeployment opportunities. At the aggregate level such targeted, and more efficient skill training might reduce long term unemployment. We also believe that information on redeployment opportunities should be provided to students to enable them to make informed career choices. This Occupation Space can also be of interest for researchers willing to analyze individual resilience in the aftermath of economic shocks, the sources of employment growth and the origins of wage inequality on the labor market. As preliminary evidence, this study shows that centrality increases individuals’ wage and reduces the risk of long-term unemployment.

## Data Availability

Access to data is limited due to confidentiality restrictions. The access was provided by the Centre d’accès sécurisé aux données -CASD. Aggregated anonymous data can be extracted upon request.

## Appendix 1: Estimating counterfactual mobility flows

We measure skill-proximity between occupations by comparing excess occupation flows with expected flows that would be only driven by observable characteristics of occupations, such as occupation’s demographic size and trend, average wage, or social composition.

The expected flows come from a regression of the number of switches observed for each of the 72,092 possible movements from occupations i to occupation j on a set of occupation characteristics. Therefore, our estimation of labor flows is based on a count variable that is always positive and of integer value. Specifically, there are 23,790 positive switches from occupation i to j. The rest of the switches are set to 0. We also set to 0 all switches for which the count was less than 10, for confidential restrictions, but also to avoid analyzing very marginal linkages. With such a dependent variable, the most appropriate regression model is a Zero-Inflated Negative Binomial regression (ZINB) which consists in two steps. The first step runs a logit regression determining the probability of observing a flow between any two pairs of occupations. The second step is a count data model estimating the number of flows from one occupation to another.

We control for observable occupations’ characteristics, including the size of both occupations in log (*lEmpi* and *lEmpj*), and the growth rate of employment in each occupation over 20032015 (*lDeltaEmp*) to capture expanding or declining trends in occupations’ demography that would explain labor flows. We add the average annual wage in each occupation in log euros (*lWage*) as well as a binary variable *WagePremia* capturing a wage increase from occupation i to j that motivates labor flows. Finally, we capture the socio-composition of each occupation in two dimensions: the average age and the share of men in each origin and destination occupations, as it is reported that both gender and age influence worker’s mobility (Topel and Ward 1992). The estimated model is the following:

$$E(Fi,j|vi,vj,wij,\varepsilon i,j) = [{1 } - \pi 0(\gamma + \delta i\,lEmpi + \delta j\,lEmpj)]e^{\alpha + \beta ivi + \beta jvj + \beta iwij + \varepsilon i,j}$$

where the vectors *v*_*i*_ and *v*_*j*_ correspond to the occupation-level variable *lEmp lDeltaEmp* and *lWage*. *wij* is the occupation-pair level variables *WagePremia*_*ij*_. *π*_0_ is the probability of *F*_*ij*_ = 0. Results are reported in Table

**Table 6:** Zero inflated negative binomial model

	Occupation movement from occupation i to j
Number of employees in occ. i (log)	0.437 (50.36)***
Number of employees in occ. j (log)	0.491 (52.50)***
Wage premium in occ j	0.056 (2.69)***
Average wage in occ. i (log)	0.167 (5.62)***
Average wage in occ. j (log)	0.199 (6.54)***
Average age in occ. i	− 0.01 (3.44)***
Average age in occ. j	− 0.005 (1.71)*
Share of men in occ. i	− 0.06 (1.79)*
Share of men in occ. j	− 0.044 (1.29)
Growth rate of employment share in occ. i	0.00 (0.11)
Growth rate of employment share in occ. j	− 0.00 (0.00)
Both urban occupations	0.085 (1.19)
Both rural occupations	0.122 (1.89)*
constant	− 7.762 (30.59)***
Observations	72,092

**p* < 0.1; ***p* < 0.05; ****p* < 0.01

Labor flow is the cumulative flow of labor from the occupation of origin (occupation i) to the destination occupation (occupation j) from 2003 to 2015 that are derived from the DADS Panel database cleaned as explained above. All other variables are constructed from the same the DADS Panel

6.

The model confirms that the volume of employment in each occupation largely explains both incoming and outcoming flows. In addition, wage premium increases the actual labor flows between occupations, while average age reduces the occupations’ mobility. Finally, the employment growth of the occupation does not influence the job switches. We defined as the “predicted” flows $\widehat{F}$_i,j_, the predictions of the ZINB model, although, we know them to be flawed, precisely because they do not consider skill-proximity. The gap between these predictions and the actual flows should therefore capture skill-relatedness of occupations.

## Appendix 2: Alternative centrality measures

We report the result of our estimations using alternative measures of centrality, namely betweenness and weighted eigenvector centrality. Betweenness captures the frequency of each node to be among shortest path between any other nodes’ pair. As said above, we don’t believe it to be a proper measure of the potential of job mobility, moreover because it is an unweighted measure. Weighted Eigenvector centrality is a generalization of Bonacich (1972) eigenvector centrality, that allows to take into account the centrality of neighbors in each nodes’ centrality scores, but to weighted networks (Newman 2004).

Table 7 reports the correlation between these alternative measures of centrality and the workers’ wage, with and without fixed effects, while Table 8 displays the result for the unemployment duration. Betweenness centrality is negatively associated with wage, failing at capturing the workers’ bargaining power. Eigenvector centrality shows expected results for the wage premia. Yet, because of capturing indirect centrality, it fails at being significantly associated with a reduction of unemployment duration, while betweenness score shows an expected results once controlling for individual fixed effects. Overall the results are less satisfying than using the Opsahl and al. (2010) centrality measure.

**Table 7:** Alternative centrality measures and wage premia

	OLS	OLS-FE	OLS	OLS-FE
	Wage	Wage	Wage	Wage
Betweenness	− 0.004	− 0.003		
	(4.26)***	(0.84)		
Eigenvector centrality (weighted)			0.018	0.008
			(17.84)***	(2.33)**
Age	0.006	0.028	0.006	0.028
	(53.16)***	(23.59)***	(53.52)***	(23.58)***
Diploma: (ref: higher diploma)				
Some college	− 0.240	− 0.112	− 0.238	− 0.111
	(61.96)***	(3.37)***	(61.45)***	(3.35)***
Upper general high-school	− 0.469	− 0.182	− 0.468	− 0.181
	(126.28)***	(4.93)***	(126.06)***	(4.91)***
Upper technical high-school	− 0.618	− 0.352	− 0.616	− 0.352
	(180.09)***	(8.37)***	(179.56)***	(8.37)***
Lower high-school	− 0.640	− 0.645	− 0.638	− 0.644
	(138.62)***	(13.77)***	(138.18)***	(13.76)***
No diploma	− 0.833	− 0.524	− 0.829	− 0.524
	(225.37)***	(11.14)***	(223.72)***	(11.13)***
Female (ref: male)	− 0.350		− 0.346	
	(171.83)***		(172.01)***	
Seniority	0.001	0.000	0.001	0.000
	(109.64)***	(1.21)	(109.31)***	(1.25)
Constant	7.448	6.483	7.240	6.380
	(976.11)***	(37.46)***	(604.30)***	(36.34)***
Year fixed effects	Yes	Yes	Yes	Yes
*R*^2^	0.30	0.01	0.30	0.01
*N*	277,479	277,479	277,479	277,479

**p* < 0.1; ***p* < 0.05; ****p* < 0.01

**Table 8:** Alternative centrality measures and unemployment duration

	OLS	OLS-FE	OLS	OLS-FE
	Wage	Wage	Wage	Wage
Betweenness	0.003	− 0.058		
	(0.61)	(1.72)*		
Eigenvector centrality (weighted)			− 0.008	0.055
			(1.27)	(1.61)
Age	0.008	0.785	0.008	0.784
	(14.32)***	(48.13)***	(14.31)***	(48.12)***
Diploma: (ref: higher diploma)				
Some college	− 0.057	− 0.099	− 0.057	− 0.126
	(2.00)**	(0.24)	(1.99)**	(0.30)
Upper general high-school	0.008	− 0.567	0.008	− 0.586
	(0.32)	(1.11)	(0.32)	(1.14)
Upper technical high-school	0.062	− 0.510	0.061	− 0.564
	(2.68)***	(0.86)	(2.66)***	(0.95)
Lower high-school	0.117	− 0.469	0.117	− 0.536
	(4.17)***	(0.76)	(4.17)***	(0.87)
No diploma	0.196	− 0.571	0.195	− 0.613
	(8.52)***	(0.93)	(8.46)***	(1.00)
Female (ref: male)	0.013		0.012	
	(1.10)		(0.95)	
constant	0.965	− 25.484	1.063	− 26.326
	(18.56)***	(32.83)***	(14.42)***	(31.77)***
Year fixed effects	Yes	Yes	Yes	Yes
*R*^2^	0.02	0.20	0.02	0.20
*N*	25,502	25,502	25,502	25,502

**p* < 0.1; ***p* < 0.05; ****p* < 0.01

## Abbreviations

CI: Confidence interval
DADS: *Déclaration Annuelles de Données Sociales* (annual declaration of social data). French administrative survey on firm-level workforce composition
EDP: Echantillon démographique permanent (permanent demographic sample)
FE: Fixed effects
LFS: Labor force survey
OLS: Ordinary least squares
PCS–ESE: Profession et Catégorie Socio-professionnelle–Emploi Salarié d’entreprise (classification of occupations and socio-profesionnal categories for private firms employees)
ZINB: Zero inflated negative binomial model

## Footnotes

1 Worker redeployment possibilities largely depends on the specificity of human capital and the possibility to use a variety of skills including reasoning, mathematical and language development in different industries or occupations (Poltaev and Robinson 2008). From this observation, the literature has used information on workers mobility across industries to identify those that offer a related set of skills allowing for more redeployment possibilities across them (Neffke and Henning 2013; Neffke et al. 2017).

2 At the three-digit level, 63.8% of workers have changed occupation at least once over the period 2003–2015, whether they are still employed in the same firm or not (Joyez et al. 2021). This figure should escalate in the aftermath of Europeans lockdowns during the COVID-19 pandemic, since a record number of workers expressed their desire to change occupation (*Baromètre de la formation et de l’emploi*, 2nd edition).

3 Occupation redeployment possibility can be a highly desired asset by giving workers more outside options on the labor market which should raise their bargaining power and their resilience in case of job displacement and thereby should influence their wage and unemployment duration.

4 Projecting bi-partite networks is often done in economic geography in order to reveal specialization patterns and the geographic proximity between either goods, occupations, patents, or research areas, using the frequency of co-localization (Balland and Rigby 2017; Guevara et al. 2016; Hidalgo and Hausmann 2009; Muneepeerakul et al. 2013).

5 The algorithm is accessible from the nw_projection Stata command, available on the Boston College Statistical Software Components (SSC) archive (Joyez 2020).

6 Since our panel is not perfectly balanced, we allow for a period of unemployment or inactivity between two occupations for a maximum of one year. In addition, we only account for occupational mobility for workers who did not change their place of living. Indeed, measuring occupational mobility without controlling for location might not be a good answer for our analysis as there exists out commuting or outmigration that would capture individual resilience and not regional resilience.

7 Edges weight below 1 initially reflect occupation dissimilarity, and therefore should be discarded from the centrality analysis of the next session.

8 Our network differs from the “occupation space” built by Muneepeerakul et al. (2013), since theirs comes from the co-location network of occupations within cities. The resulting proximity between occupations reflects thus the geographical proximity of jobs, whereas in ours the proximity reflects the skill-proximity between occupations.

9 Disparity is defined for node *i* as the index $Y (i) = {\sum }_{j} [\frac{{w}_{ij}}{{s}_{i}}]^{2}$, where *w*_*ij*_ and *s*_*i*_ stand respectively for edges’ weight and nodes’ strength (see Barthélemy et al. 2005).

10 See Joyez (2017) for a weighted generalization of Freeman (1978) degree centralization index.

11 In Appendix 2 we report our estimations’ results based on two other centrality indexes, namely centrality and weighted eigenvector centrality. As expected, they fail at capturing the immediate outside option of workers, contrary to the centrality index suggested by Opsahl et al. (2010).

12 An interative map of the network can be found at https://cjoyez.github.io/OccupationSpace/network/#

13 Category (ii) corresponds to a diploma that is called “BTS” (Brevet Technicien Supérieur). Category (iii) corresponds to a diploma that is called “Baccalauréat general”. Category (iv) corresponds to a diploma that is called BEP/CAP. Category (v) corresponds to a diploma that is called “Brevet des collèges”.

14 We focus on the sample of workers with a fixed employment contract. Namely we focus on workers with a CDD “contrat à durée déterminée” and CDI “contrat à durée indeterminés” which are the two principal permanent employment contracts offered on the French labor market.

15 Our data allows to follow workers for 6 consecutive trimesters.
